# On reliability of annotations in contextual emotion imagery

**DOI:** 10.1038/s41597-023-02435-1

**Published:** 2023-08-12

**Authors:** Carlos A. Martínez-Miwa, Mario Castelán

**Affiliations:** Robótica y Manufactura Avanzada, Centro de Investigación y de Estudios Avanzados del Instituto Politécnico Nacional, Ramos Arizpe, Coahuila 25900 México

**Keywords:** Human behaviour, Computer science

## Abstract

We documented the relabeling process for a subset of a renowned database for emotion-in-context recognition, with the aim of promoting reliability in final labels. To this end, emotion categories were organized into eight groups, while a large number of participants was requested for tagging. A strict control strategy was performed along the experiments, whose duration was 13.45 minutes average per day. Annotators were free to participate in any of the daily experiments (the average number of participants was 28), and a *Z*-Score filtering technique was implemented to keep trustworthiness of annotations. As a result, the value of the agreement parameter Fleiss’ Kapa increasingly varied from slight to almost perfect, revealing a coherent diversity of the experiments. Our results support the hypothesis that a small number of categories and a large number of voters benefit reliability of annotations in contextual emotion imagery.

## Introduction

Generally, image based Machine Learning (ML) techniques focus on objects of the world. This kind of data is objective in nature, as it does not depend on personal opinions. Data annotation related to these applications is rather unambiguous, as consensus among labelers is not necessarily required, i.e., there is no controversy in the multiple categories present in the world, such as chair, human, dog, etc. One of the most renowned datasets in ML community is ImageNet^1^, which consists of 3.2 million annotated images, spanning 5,247 object categories. The creators of ImageNet believe that “a large-scale ontology of images is a critical resource for developing advanced, large-scale content-based image search and image understanding algorithms, as well as for providing critical training and benchmarking data for such algorithms”. This *the more, the better* approach has gained popularity in recognition tasks, although questions related with the world of the subjective arise when results in emotion recognition are not as sound as their object recognition counterpart.

Earlier works on emotion datasets were based on people’s facial expressions. A clear example is the Japanese Female Facial Expression database^2^. JAFFE consists of 219 images of 10 people posing 3 to 4 examples of six basic emotions: anger, surprise, disgust, enjoyment, fear, and sadness, along with neutrality. This dataset achieved a rank correlation between Gabor model and human data of 67.9%. Other well-known database examples are: Maja and Michel Initiative, better known as MMI^3^, with 79 sets of facial expressions from 25 participants and six basic emotions; Multi-Pie^4^, yielding approximately 750,000 images of 337 people and seven emotions; and the Cohn-Kanade dataset (CK)^5^, with 486 sequences of 97 subjects, captured under both static and dynamic lab conditions. In the Extended Cohn-Kanade Dataset (CK+)^6^, an enhanced version of the Cohn-Kanade, 123 persons were recorded posing six basic emotions plus contempt for a total of 593 images. Unlike JAFFE, CK+ has both gender and nationality participants, offering greater diversity. CK+ integrates, also, a smaller portion of non-posed emotion images, i.e., moments when subjects were smiling within trials. This database yields very good results with a 94.5% in action units (AU) detection, and even 100%, in some cases, for emotion recognition; nonetheless, it still relies on people’s facial features. Some other works that focus on spontaneous emotions are Belfast^7^, Denver Intensity of Spontaneous Facial Action (DISFA)^8^, and the Affectiva-Mit Facial Expression Dataset (AM-FED)^9^. The Facial Emotion Recognition 2013 (FER-2013) dataset was built by Pierre Luc Carrier and Aaron Courville^10^. This database significantly increases the number of images, reaching 35,887 human face snapshots. The set of emotions involved are like those previously detailed, that is, six basic emotions coupled with neutrality. In this dataset, image conditions are no longer controlled, as Google images that matched a set of 184 emotion words were used. FER-2013 reached a 71.16% as it highest accuracy in the facial expression recognition challenge 2013. Nevertheless, despite these results and their significantly high account, the acquired images continue to show only people’s faces.

As noted, algorithms tested on this kind of facial related data exhibit good results. Nonetheless, many databases are developed in controlled conditions, so captured emotions are not entirely genuine. As a result, databases which consider not only the subjects, but also their context, arose. Acted Facial Expressions in the Wild (AFEW)^11^ gathers 957 videos from 37 films, with 220 individuals ranging in age from 1 to 70 years. Seven emotions are contemplated: six basic emotions and neutrality. Although AFEW illustrates more realistic emotions, these are still acted since they come from performers. Consequently, annotations may be biased as most people feel a degree of sympathy or rejection for certain actors. Furthermore, images were labeled by a single individual. This means that annotations whose reliability and agreement cannot be measured (for example, using Fleiss’ Kappa^12^) are taken as true.

Recently, Context-Aware Emotion Recognition (CAER)^13^ was introduced. This database considers two modalities: (1) 13,201 videos from 79 different TV shows, ranging from 30 to 120 frames each; and (2) 70,000 images extracted from such videoclips. As an overall, both modalities depict six emotions: anger, disgust, fear, happiness, sadness, surprise and neutral. Annotations were carried out by six individuals and then evaluated by other three. If two or more annotators labeled a video with the same categories, the videoclip was considered valid. Even when the number of taggers was increased with regard to previous approaches, images were still selected from movies, meaning that the context of the scene is not necesarily emphasized, i.e., the main performer is always on foreground. Additionally, taggers may have previously developed feelings toward certain movies, scenes or performers.

A widely used database is EMOTions In Context or EMOTIC^14^. This image set is made up of 23,571 images from MSCOCO^15^, Ade20k^16^ and Google with a total of 34,320 annotated examples (subjects) along the whole dataset. EMOTIC combines two emotion representations. The first one is based on discrete categories, i.e., 26 different emotions collected from a 400-word vocabulary of affective states. The second one involves continuous dimensions by resorting to the VAD (Valence, Arousal, Dominance) Emotional State Model^17^. The amount of scorers in EMOTIC is as follows: validation set (10% of images): from 2 to 16 scorers; test set (20% of images): from 0 to 5 scorers; and training set (70% of images): 1 scorer. While EMOTIC considerably increases the maximum number of labelers, there is a clear imbalance for annotated emotions across the different subsets. As the training set was annotated by only 1 person, it is not possible to validate such votings. EMOTIC is probably the most challenging database to date. The subjects depicted within its images are not only facing forward, but may be looking to the side, or even backward; they may be near or far away, or even partially occluded.

**Fig. 1:**
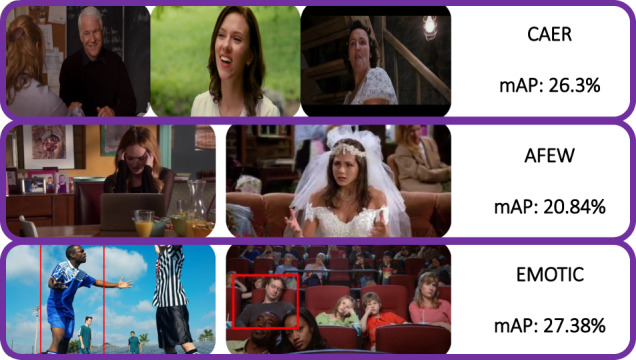
Examples of images from context aware datasets: CAER^13^ at top row, AFEW^11^ in the middle, and EMOTIC^14^ at the bottom row. It is to note how, despite comprising a high number of images, mean average precision (mAP) remains low, which may in turn be due to a low reliability in annotations.

Examples of images in some of the datasets described above are shown in Fig. 1. Note how, unlike CAER and AFEW, EMOTIC includes images with greater focus on the context, translating into potentially more complex annotations.

Emotions are more related to the field of the Humanities than to the field of Engeneering, where many annotators may come from. While one person may see joy in a subject, another person may perceive different, even opposite emotions. People have difficulty either acknowledging or naming what they feel. Brown^18^ interviewed 7,500 subjects and asked them to share a personal experience, and to recognize and name the emotions they were experiencing at the time. On average, just three emotions were elicited: happiness, sadness and anger, revealing the limited vocabulary of most humans for identifying and communicating feelings. Other studies have confirmed difficulty for people when recognizing their own emotions^19,20^. This phenomenon endows the emotion recognition problem with significant ambiguity. To alleviate this issue, the focus has been on evaluating the degree of attention of taggers through control images, although other important factors may not be taken into account, for example, the number of images to be labeled on a daily basis.

**Fig. 2:**
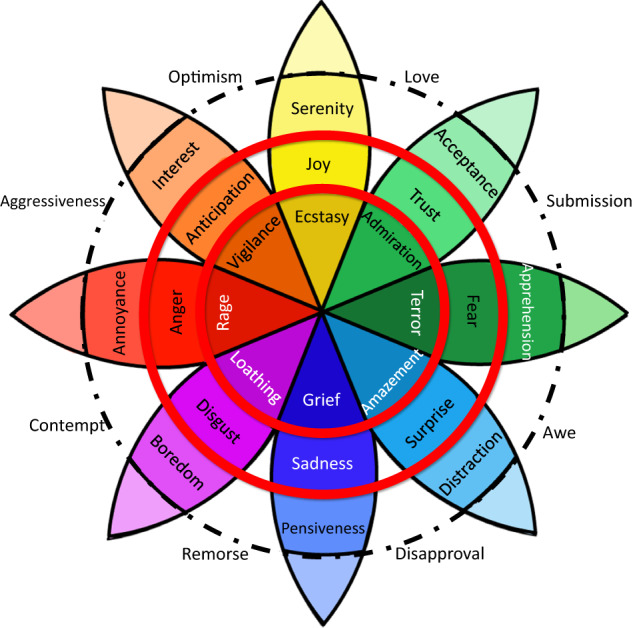
Psychoevolutionary theory of emotion. The eight basic emotions defined by Plutchik appear inside the solid circles. Just as colors, emotions are grouped according to their similarity and strength. A lighter color saturation represents basic emotions with a lower intensity. A stronger saturation is associated with basic emotions and greater intensity. Finally, the emotions located on white background are linked to combinations of contiguous emotions.

As a consequence of excessive annotation journeys, annoyance, boredom or tiredness of annotators, the obtained labels may lack accuracy. Notwithstanding, even if the attentiveness of annotators was controlled, their labelings might not be entirely reliable for the learning of computer algorithms. To set an example, let us take the model proposed by Plutchik^21^ in his psychoevolutionary theory of emotion, where a cone-shaped model is introduced. By means of a color code, Plutchik suggests that emotions combine in a similar way as colors (Fig. 2). Additionally, this model groups emotions by similarity, placing each emotion 180° from its opposite. If we took into account this arrangement for emotion labeling (and allowing for multi-tagging), any subject could assign more than four categories to a single image. In this case, the probability of having opposite annotations would be high, and such annotations would become unreliable. Therefore, apart from promoting attentiveness, it is necessary to guarantee reliability of annotations, i.e., to preserve only the most representative labels.

In this paper, we approach these concerns by performing experiments on relabeling a subset of the challenging emotion recognition dataset, EMOTIC^14^. We introduce an attentiveness promotion (AP) strategy for encouraging accuracy in annotators and propose the *Z*-Score threshold to filter out inconsistent labels. We focus on the impact of considering a large number of labelers and a reduced amount of categories, therefore, the scope or our work does not include artificial emotion recognition performance.

The contents of this paper are presented as follows. Section “Methods” details the labeling strategy performed on a subset of EMOTIC. In addition, this section describes the proposed methodology for promoting attentiveness in annotators (AP), along with a filtering technique based on the *Z*-Score designed to keep only those annotations with the highest representativeness. The outcome of the proposed strategies are provided in Section “Results”. Finally, derived conclusions and perspectives are shared in Section “Discussion”.

## Methods

### Emotion recognition annotation protocol

We performed a reannotation process on a subset of EMOTIC dataset since it is one of the most used and cited to date^14,22–26^. Our method comprised four main stages: (1) image subset selection; (2) image subset annotation; (3) application of first control (preserving annotations from attentive subjects) and (4) application of second control (*Z*-Score filter for reliability of annotations). All methods were developed through MATLAB 2022 (MathWorks, Inc., Natick, MA, USA), and the scripts were generated by the main author. Our code and annotations are included in the resulting dataset^27,28^.

### Image subset selection

Very few EMOTIC images correspond to a single category, i.e., the classification is not only a multi-class problem, but also a multilabel one, leading to possible disperse results. Some of these discrete categories are specific and not common, as stated in Heredia *et al*.^24^. Selecting from a large number of categories for tagging could be exhausting for labelers, possibly leading to less attention to the process. We regrouped the original 26 discrete emotion categories of EMOTIC into a smaller number of classes, as per Heredia *et al*.^24^ In this way, similar emotions were clustered into the eight Plutchick’s groups, as detailed in Table 1.

**Table 1:** EMOTIC rearrangement into the eight main emotions proposed by Plutchik, as in the work of Heredia *et al*.^24^.

Plutchik’s category	Emotions originally labeled in EMOTIC
Anger	Anger, Annoyance, Disapproval
Anticipation	Anticipation, Engagement
Disgust	Aversion, Disconnection, Fatigue, Yearning
Fear	Disquitement, Embarrassment, Fear
Joy	Affection, Excitement, Happiness, Pleasure
Sadness	Pain, Sadness, Sensitivity, Suffering
Surprise	Doubt/Confussion, Suprise
Trust	Confidence, Esteem, Sympathy, Peace

One issue in EMOTIC is the imbalance in the number of images per annotated emotion (see Table 2). For example, *Anticipation* accounts for 52.38%, 26.54% and 32.42% of validation, test, and training sets, respectively. Similarly, the second and third categories with the greatest number of images are *Joy* and *Trust*; *Joy* holding for 32.04% validation, 32.02% test and 29.06% training, while *Trust* holding for 5.85% validation, 20.59% test and 20.21% training. In comparison, the rest of the categories present a very low percentage of corresponding images. In this regard, for building the relabeling subset, a selection criterion that homogenizes the number of images per group was developed. To this end, we used the original EMOTIC’s labels. For every image, the percentage of each emotion with respect to the number of labelers was calculated (Eq. 1). The decision to choose the percentage instead of the Fleiss’ Kappa was motivated by the imbalance in the number of voters in EMOTIC’s original annotations. The emotion with the maximum percentage of incidence was taken as its most representative through:

1 $$Pc{t}_{Emotion}=\frac{{\rm{N}}{\rm{u}}{\rm{m}}{\rm{b}}{\rm{e}}{\rm{r}}\;{\rm{o}}{\rm{f}}\,{\rm{v}}{\rm{o}}{\rm{t}}{\rm{e}}{\rm{s}}\;{\rm{p}}{\rm{e}}{\rm{r}}\;{\rm{e}}{\rm{m}}{\rm{o}}{\rm{t}}{\rm{i}}{\rm{o}}{\rm{n}}}{{\rm{T}}{\rm{o}}{\rm{t}}{\rm{a}}{\rm{l}}\;{\rm{n}}{\rm{u}}{\rm{m}}{\rm{b}}{\rm{e}}{\rm{r}}\;{\rm{o}}{\rm{f}}\,{\rm{v}}{\rm{o}}{\rm{t}}{\rm{e}}{\rm{s}}}.$$

**Table 2:** Total number of EMOTIC’s images after being rearranged into eight emotion categories, in accordance to EMOTIC’s original annotations.

Plutchik’s category	Validation	Test	Training	Total
Anger	78	300	702	1,080
Anticipation	1,747	1,932	7,450	11,129
Disgust	134	530	2,170	2,2834
Fear	29	234	610	873
Joy	1,068	2,331	6,575	9,974
Sadness	54	243	665	962
Surprise	29	210	744	983
Trust	195	1,499	4,790	6,484
TOTAL	3,334	7,279	23,706	34,319

Note how *Anticipation*, *Joy* and *Trust* considerably represent the majority of images.

As a result, 140 images per emotion were selected, gathering a final subset of 1,120 EMOTIC images.

### Annotating the image subset

We requested university students to be part of the relabeling process. 206 persons, 27.18% male and 72.82% female, with an average age of 21.33 ± 4.32 years, from different educational areas and living in the same city agreed to participate. The annotation process took 20 working days, and participants were free to decide the number of days they wanted to be part of the experiment. The attendance of at least 15 people per day was monitored.

Annotations were carried out as follows: an online form was created with the selected subset of 1,120 EMOTIC images. The instrument was designed to be easy to use and user-friendly in order to motivate the participants, involve them in the test and avoid boredom. Also, the survey could be answered either on a computer or a mobile phone. All subjects were given an informed consent form indicating that their contribution was completely voluntary with no foreseeable risks, and that their personal data would be strictly confidential.

**Fig. 3:**
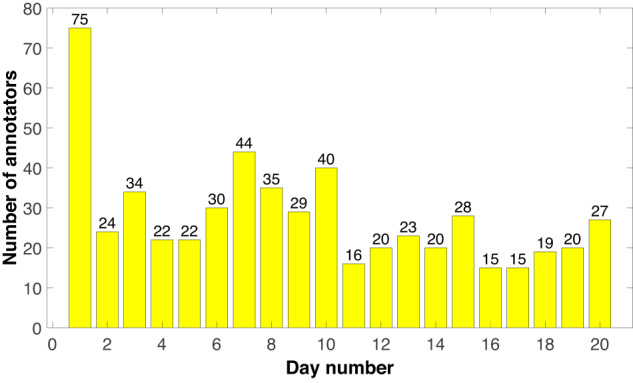
Number of annotators, from a total of 206, for each of the 20 working days of the annotation process. The minimum number of participants per day was 15. The number of images to label per day was 56. Only one emotion dyad was allowed to be labeled per day. As expected, the majority of participants attended during the first day of the experiment.

Figure 3 depicts the number of labelers per day during the annotation process. Daily, each tagger was shown 56 different images. Labelers were asked to annotate, image by image, the emotion perceived in the highlighted person, being allowed to select one dyad from a list. Table 3 presents the emotion dyads used during the labeling process. The first emotion stated in the dyad corresponds to each of the eight categories of Plutchik’s model; the second, represents a synonym extracted from the list presented by Kosti *et al*.^14^. The average response time of the experiment, per day, was 13.45 minutes.

**Table 3:** Dyads of emotional categories.

Emotional category dyads			
1:Anger/Rage	2:Engagement/Anticipation	3:Disgust/Disconnection	4:Fear/Worry
5:Joy/Affection	6:Sadness/Discouragement	7:Surprise/Amazement	8:Trust/Peace

Each category consists of two representative emotions. Labelers were allowed to select one dyad per image. Dyads were used to facilitate the selection of similar emotions belonging to the same category.

### Promoting attentiveness of annotators

**Fig. 4:**
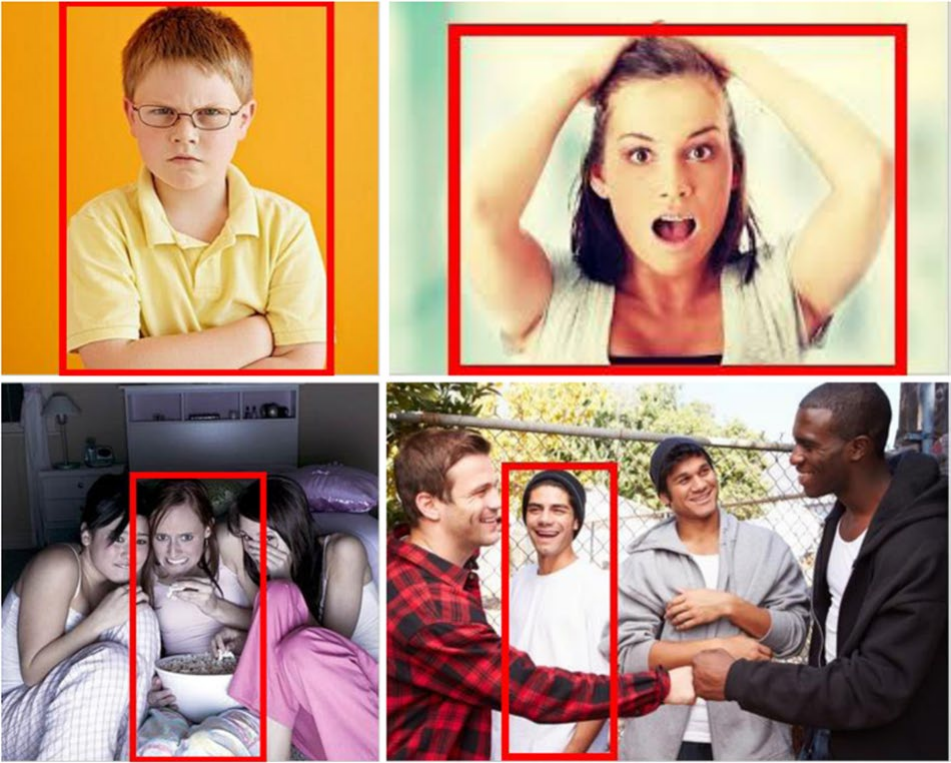
Examples of EMOTIC images selected as control images. *Anger/Rage* and *Surprise/Amazement* correspond to the top left and top right images, respectively, while *Fear/Worry* and *Joy/Affection* correspond to the bottom ones.

Control strategies are designed to monitor the votings of participants and prevent noisy annotations. Usually, these techniques include reference images and categories that were manually selected; moreover, no prior validation stages are held. In the current work, we propose AP, a strategy that consists of including, for each experiment (per day), six control images strongly representing a particular emotion. For selecting these control images, a pilot study was conducted prior to the main annotation experiments. Five participants, apart from the 206 labelers were asked to annotate the emotions they perceived in the 1,120 EMOTIC images subset. Images with at least 4 coincidences among voters were taken as control images. Figure 4 illustrates examples of these images. In the main experiment, six different control images were included daily. In addition, one repeated image out of the 56 was included. This would ensure that participants were attentive: if votes on repeated images mismatched, or if there were less than 5 correctly tagged control images, the test for that participant was discarded.

Once the relabeling phase was accomplished, the parameter Kappa was calculated (Table 4). On average, the Kappa value reached 0.42, representing a *Moderate* agreement. Regarding the number of labelers for each image, the last column in Table 4 show averages per Kappa category. The average number of annotators per image was 27.9.

**Table 4:** Number of images and mean number of annotators for each Kappa category for AP (Attentiveness Promotion).

Kappa category	Number of images	Average number of annotators
Poor	1	15
Slight	314	29.1
Fair	391	27.5
Moderate	184	28.1
Substantial	99	28.6
Almost Perfect	131	27.2

### Enhancing annotation reliability

**Fig. 5:**
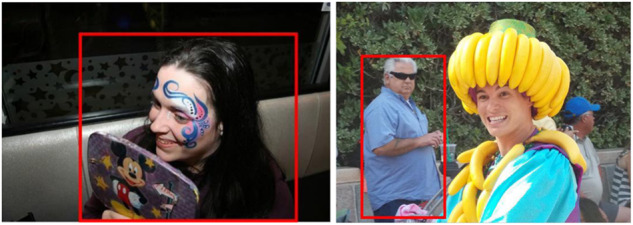
Examples of EMOTIC dataset images. For the left image, the following was obtained: 1 vote for *Anger/Rage*, 1 for *Engagement/Anticipation*, 1 for *Disgust/Disconnection*, 23 for *Joy/Affection*, 3 for *Surprise/Amazement*, 1 for *Trust/Peace*, and 0 for all other categories. For the right image, the next scores were obtained: 1 vote for *Anger/Rage*, 13 for *Engagement/Anticipation*, 7 for *Disgust/Disconnection*, 5 for *Surprise/Amazement*, 2 for *Trust/Peace*, and 0 for the remaining classes. It is to note the dispersion in the number of votes, which could be caused by the projection of emotions by annotators.

Our labeling process relies on the idea of significantly increasing the number of annotators for a reduced number of categories. Also, the number of options to be selected is restricted to one by image. Our aim is that annotations become more practical and less strenuous for labelers, possibly leading to more accurate labels. As expected, the agreement increased considerably as the number of categories decreased. However, despite AP, for some images, there were dyads that obtained very few votes compared to the dyad with the majority of votes. For example, for the left image in Fig. 5 the following was obtained: 1 vote for *Anger/Rage*, 1 for *Engagement/Anticipation*, 1 for *Disgust/Disconnection*, 23 for *Joy/Affection*, 3 for *Surprise/Amazement*, 1 for *Trust/Peace*, and 0 for the remaining categories, representing a Kappa of 0.53. If we take these facts into account, we have a multi-labeling effect containing non-significant emotions. This clear dispersion of labels includes votes for categories that are opposites in Plutchik’s model, suggesting that the labelers could be projecting their own emotions along the process, i.e., instead of registering what the person in the image might be feeling, they captured their own feelings about the context and person. Although it is important that annotations reflect honesty, such labels must be reliable for artificial learning. Consequently, it becomes necessary to implement a new type of control that not only monitors the attentiveness of annotators (AP), but that also improves the reliability of their annotations. In this sense, a Reliability Enhancement (RE) strategy based on a *Z*-Score filtering, which involves submitting every image votes to the *Z*-Score, is introduced. The standard variable *Z* indicates how many standard deviations a piece of data is away from the mean. Its formula is defined as:

2 $$Z=\frac{X-\mu }{\sigma },$$

where *X* stands for the measured value, *μ* for the expected value or mean, and *σ* for the standard deviation. Emotions with a *Z* value equal or higher than 0 were selected as new labels, while the rest was discarded. Via this new filter, a lower number of labels per image can be ensured. Without considering this filter, some images with more than four labeled categories were spotted, indicating complexity in identifying leading emotions and the possibility that some of them were opposites in Plutchik’s model, implying low reliability.

## Results

This section details the effect of applying the proposed RE filtering to the labeled data. Four quantitative aspects in which the filtering effect was evaluated are described in the next subsection, while qualitative aspects are later discussed.

### Quantitative results

#### Kappa category

After applying RE, the mean Kappa value for our subset of 1,120 images turned from 0.42 in AP to 0.64. Table 5 depicts the number of images by Kappa category with attentive annotations (AP) compared to reliable ones (RE). The reduction of images in the three lower groups, *Poor*, *Slight* and *Fair*, is noticeable. *Moderate* and *Almost Perfect*, on their own, markedly rose by 0.95 and 2.39 times, respectively. A sharp decrease is also evident in the *Substantial* group. This is caused by the *Z*-Score threshold described in the preceding section. After RE was applied, images from all different groups kept only the most reliable emotions, that is, those with the most representative votes. As a result, the Kappa value increased, shifting these images to a higher Kappa category, mainly to *Almost Perfect*.

**Table 5:** Amount of images by Kappa Category with AP (Attentiveness Promotion) and RE (Reliability Enhancement) controls.

Kappa category	AP	RE
	Number of images	Number of images
Poor	1	0
Slight	314	44
Fair	391	230
Moderate	184	358
Substantial	99	44
Almost Perfect	131	444

#### Number of labels

Another comparative feature concerns the number of labels per image. Table 6 shows the amount of images per number of labels; in other words, how many images were annotated with 1, 2, 3, …, or 8 labels. At AP stage, most of the images owned 3 to 6 labels. However, after RE, almost all images kept 1 or 2 labels, and a few more kept 3 or 4. Also, while initially there were images with 8 labels, once RE was applied labels were reduced to a maximum of 4. This is a favorable reliability indicator, as the probability of describing opposite pairs of emotions increases when more than 4 categories are labeled in the same image.

**Table 6:** Number of images after AP (Attentiveness Promotion) and RE (Reliability Enhancement) by number of categories.

Number of labels	AP	RE
1	54	444
2	114	464
3	188	176
4	254	36
5	228	0
6	179	0
7	78	0
8	25	0

The maximum number of labels is reduced from 8 to 4, suggesting improved reliablity.

#### Number of annotators

**Fig. 6:**
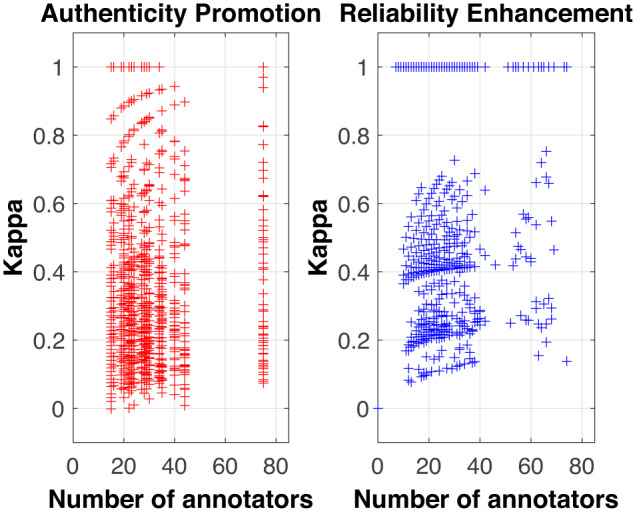
Number of annotators by the individual Kappa value obtained for each image. The different “ + ” signs represent individual images. On the left side, it is shown how, with AP (Attentivess Promotion), clusters are not clearly delimited; on the contrary, applying RE (Reliability Enhancement) reveals more separable groups, ranging from 0.15 to 0.3, 0.4 to 0.6, and one with a value of 1.

The Kappa coefficient per number of annotators was computed for every image (Fig. 6). As per AP labels, a higher concentration of images in the range of 0.05 to 0.5 occurs, representing *Slight* to *Moderate* Kappa values. Once RE is applied, values of Kappa exhibit a better delimited distribution with clusters ranging from 0.15 to 0.3, from 0.4 to 0.6 and a cluster whose only value is 1; these three clusters reveal greater separability among *Fair*, *Moderate* and *Almost Perfect* Kappa values. These results confirm those of Table 5, where three Kappa categories contain the most noteworthy scores after applying RE.

Additionally, Table 7 lists the mean Kappa for AP and RE, respectively. Based on AP, a Kappa of 0.37 was achieved representing a *Fair Agreement*. In contrast, after applying RE, the value of Kappa rose to 0.66, corresponding to a *Substantial Agreement*.

**Table 7:** Mean Kappa after AP (Attentiveness Promotion) and RE (Reliability Enhancement).

	Mean Kappa
AP	0.37 (Fair agreement)
RE	0.66 (Substantial agreement)

#### Labeled emotion

**Fig. 7:**
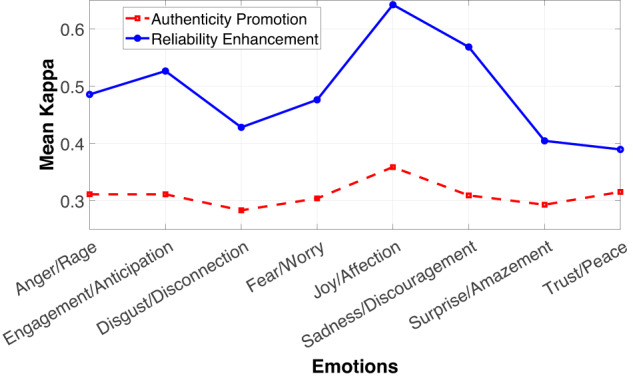
Mean Kappa for each dyad category. It is noteworthy how, for AP, Kappa remains almost uniform for all emotions (red line). On the contrary, after applying RE (blue line), there are clear boundaries between most emotions, with *Joy/Affection*, *Sadness/Discouragement*, *Anger/Rage* and *Engagement/Anticipation* standing out.

The average Kappa for each emotion was evaluated and is shown in Fig. 7. From the visual analysis of the figure, it is remarkable how, by relying on attentivenes control, the distribution of labels is practically uniform (K~0.3), with only the category *Joy/Affection* standing out. This indicates that there was little or no agreement among people to distinguish emotions, reinforcing what is suggested by Spielberg and Reheiser^19^, Alia *et al*.^20^, and Brown^18^.

After assessing and improving reliability of annotations, a better arrangement among categories was obtained, emphasizing *Joy/Affection*, followed by *Sadness/Discouragement* and *Engagement/Anticipation*, with mean Kappa values of 0.7, 0.65 and 0.6, correspondingly. This new configuration suggests that, with fewer categories to label, annotators are more capable of discerning between the emotions they perceive in the subjects highlighted within the image.

### Qualitatively

**Fig. 8:**
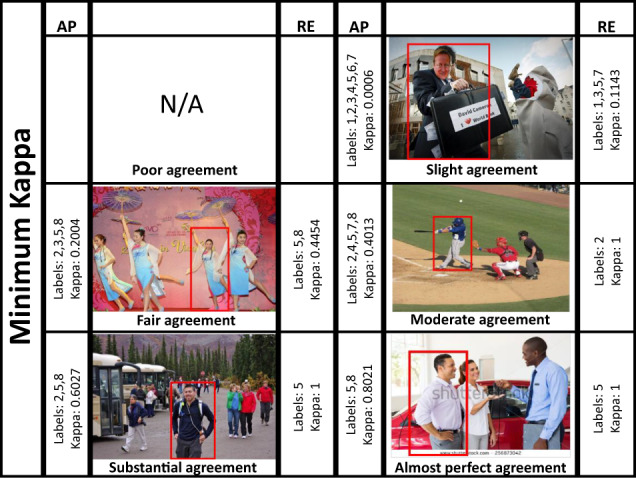
Images with minimum Kappa at every Kappa category (AP: Attentiveness Promotion, RE: Reliability Enhancement, N/A: Not Available). On each side, the corresponding labels and Kappa are shown (left for attentiveness and right for reliability evaluation). It is noteworthy that, after RE, the number of categories decreased, while the Kappa increased. Moreover, under visual inspection, the prevailing categories are more representative than the previous ones, thus more trustworthy. Numbers stand for: 1:*Anger/Rage*, 2:*Engagement/Anticipation*, 3:*Disgust/Disconnection*, 4:*Fear/Worry*, 5:*Joy/Affection*, 6:*Sadness/Discouragement*, 7:*Surprise/Amazement*, 8:*Trust/Peace*.

**Fig. 9:**
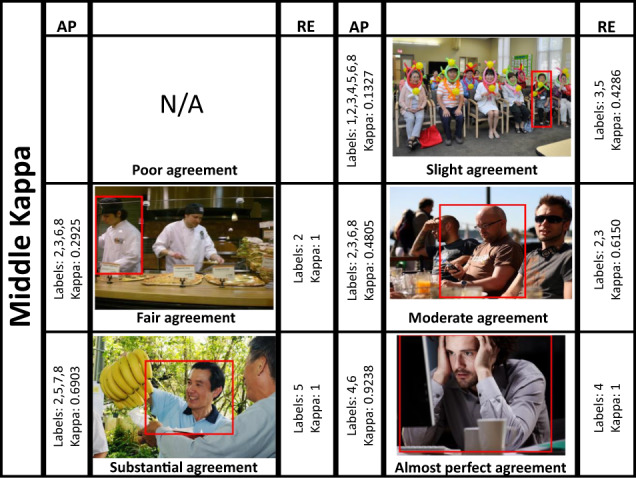
Images with middle Kappa at every Kappa category (AP: Attentiveness Promotion, RE: Reliability Enhancement, N/A: Not Available). On each side, the corresponding labels and Kappa are shown (left for attentiveness and right for reliability evaluation). It is noteworthy that, after RE, the number of categories decreased, while the Kappa increased. Moreover, under visual inspection, the prevailing categories are more representative than the previous ones, thus more trustworthy. Numbers stand for: 1:*Anger/Rage*, 2:*Engagement/Anticipation*, 3:*Disgust/Disconnection*, 4:*Fear/Worry*, 5:*Joy/Affection*, 6:*Sadness/Discouragement*, 7:*Surprise/Amazement*, 8:*Trust/Peace*.

**Fig. 10:**
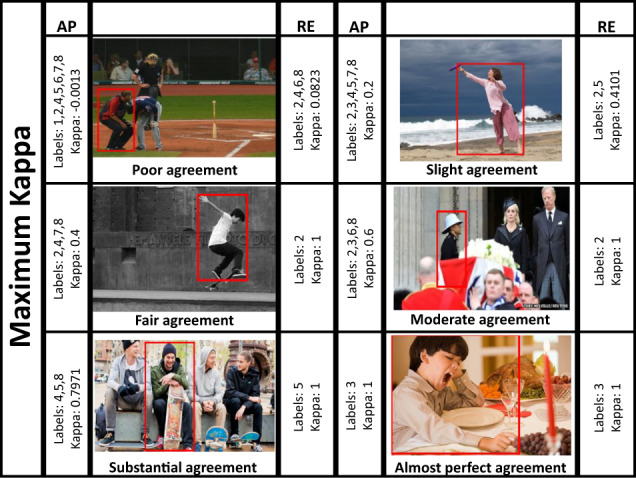
Images with maximum Kappa at every Kappa category (AP: Attentiveness Promotion, RE: Reliability Enhancement). On each side, the corresponding labels and Kappa are shown (left for attentiveness and right for reliability evaluation). It is noteworthy that, after RE, the number of categories decreased, while the Kappa increased. Moreover, under visual inspection, the prevailing categories are more representative than the previous ones, thus more trustworthy. Numbers stand for: 1:*Anger/Rage*, 2:*Engagement/Anticipation*, 3:*Disgust/Disconnection*, 4:*Fear/Worry*, 5:*Joy/Affection*, 6:*Sadness/Discouragement*, 7:*Surprise/Amazement*, 8:*Trust/Peace*.

Finally, a qualitative comparison of image annotations relying on AP and after applying RE is presented. Images that reached the maximum, median and minimum Kappa for each category were selected and annotated emotions were extracted. Table 8 shows these results. It is noticeable that, for all images, the Kappa increased as a consequence of the reduction in the amount of labels. For instance, the image with *Poor* agreement based only on AP, reduced from 7 to 4 categories after RE. Consequently, its Kappa rose from −0.001 to 0.08, i.e., from *Poor* to *Slight* agreement. In the same way, images with *Fair*, *Moderate* and *Substantial* agreements decreased from 3 and 5 categories, to only 1 or 2, i.e., their Kappa turned to *Almost Perfect* agreement in most cases. This behavior is supported and can be appreciated in a visual way through the images in Figs. 8–10.

**Table 8:** Labels and average Kappa value for images with maximum, medium and minimum Kappa at the different Kappa categories.

AP Kappa category	AP min. Kappa	Obtained labels for min. Kappa	AP med. Kappa	Obtained labels for med. Kappa	AP max. Kappa	Obtained labels for max. Kappa
Poor	—	—	—	—	−0.0013	1,2,4,5,6,7,8
Slight	0.0006	1,2,3,4,5,6,7	0.1327	1,2,3,4,5,6,8	0.2	2,3,4,5,7,8
Fair	0.2004	2,3,5,8	0.2925	2,3,6,8	0.4	2,4,7,8
Moderate	0.4014	2,4,5,7,8	0.4805	2,3,6,8	0.6	2,3,6,8
Substantial	0.6027	2,5,8	0.6903	2,5,7,8	0.7971	4,5,8
Almost Perf.	0.8021	5,8	0.9238	4,6	1	3
**RE Kappa category**	**RE min. Kappa**	**Obtained labels for min. Kappa**	**RE med. Kappa**	**Obtained labels for med. Kappa**	**RE max. Kappa**	**Obtained labels for max. Kappa**
Poor	—	—	—	—	0.0823	2,4,6,8
Slight	0.1143	1,3,5,7	0.4286	3,5	0.4101	2,5
Fair	0.4454	5,8	1	2	1	2
Moderate	1	2	0.6150	2,3	1	2
Substantial	1	5	1	5	1	5
Almost Perf.	1	5	1	4	1	3

The upper section shows the results of evaluating labels after AP. The bottom section displays the scores for the same images used in the top section, but after applying RE. Numbers stand for 1:*Anger/Rage*, 2:*Engagement/Anticipation*, 3:*Disgust/Disconnection*, 4:*Fear/Worry*, 5:*Joy/Affection*, 6:*Sadness/Discouragement*, 7:*Surprise/Amazement*, 8:*Trust/Peace*. (AP: Attentiveness Promotion, RE: Reliability Enhancement; min.: minimum; med.: median; max.: maximum).

**Fig. 11:**
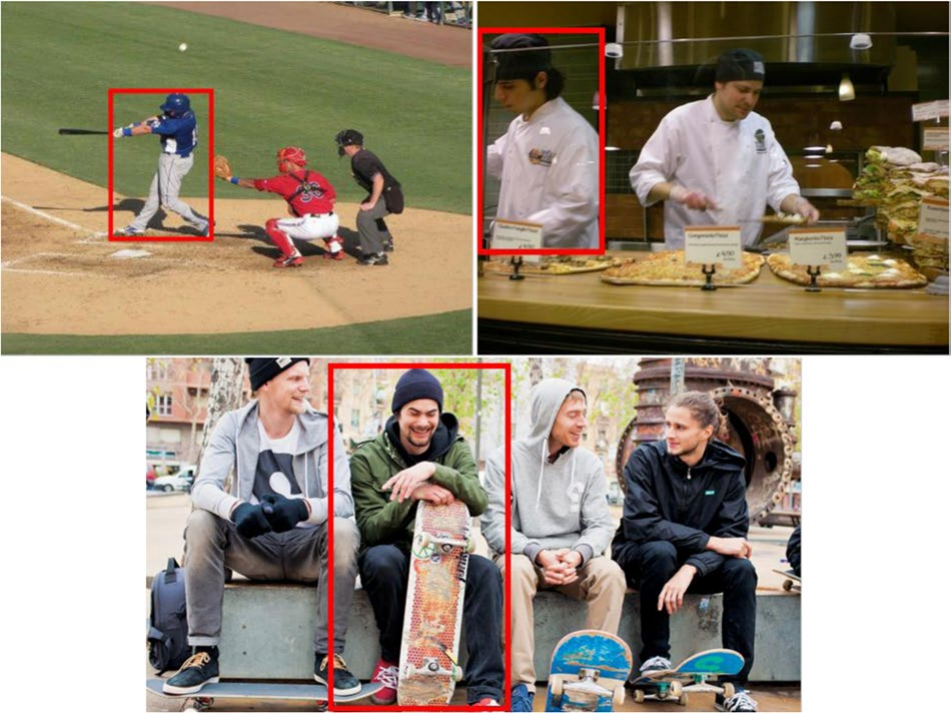
Examples of EMOTIC images with a minimun (top left), medium (top right) and maximum Kappa (bottom). Upon AP (Attentiveness Promotion), these images involved multiple categories labeled. However, some emotions may not be entirely related to the content of the image. For instance, the top left image was labeled with categories *Engagement/Anticipation*, *Fear/Worry*, *Joy/Affection*, *Surprise/Amazement* and *Trust/Peace*. The top right image included classes *Engagement/Anticipation*, *Disgust/Disconnection*, *Sadness/Discouragement* and *Trust/Peace*. Lastly, the image below featured *Fear/Worry*, *Joy/Affection* and *Trust/Peace*. After RE (Reliability Enhancement), unrelated emotions were removed maintaining the most representative ones: 2 for the top images and 5 for the last one.

The new labels seem to provide a more realistic picture of the annotated emotions. This can be analyzed in Fig. 11. For example, evaluating AP, the top left image in the figure includes the dyads *Engagement/Anticipation*, *Joy/Affection*, *Fear/Worry*, *Surprise/Amazement* and *Trust/Peace*. If we look closely, the remarked subject is playing baseball. Thus, the emotion *Engagement/Anticipation*, qualitatively, is the one that corresponds the most with the image and its context. The top right image in Fig. 11 encompasses the classes *Engagement/Anticipation*, *Disgust/Disconnection*, *Sadness/Discouragement* and *Trust/Peace* previous to RE. Paying attention to the person marked in red, *Engagement/Anticipation* emerges as the most related category. Finally, the bottom image comprised *Fear/Worry*, *Joy/Affection*, along with *Trust/Peace* before applying RE. From the image it is clear that the highlighted subject is socializing, even smiling. Therefore, the emotion *Joy/Affection* has the strongest association with the image. Thanks to the reliability improvement process based on RE, it was possible to preserve only the most trustworthy categories, i.e., those that best matched the corresponding images and discard less related emotions that could be more confusing or ambiguous.

## Discussion

Emotion recognition has attracted the attention of countless applications within computer vision, such as security, and communication, to name a few. To accomplish this task, the creation of databases that accurately represent emotions is necessary. A large number of datasets have been presented in the literature, from the ones that focus on facial expressions, to those that contemplate environmental features. Some capture only individuals’ faces, omitting other elements. This sort of images is, for the most part, developed in controlled conditions, so the expressions collected are not genuine.

Contrary to object detection, emotion recognition is a subjective task. Hence, emotion recognition datasets are susceptible to annotators’ interpretation. So far, methods on emotion detection focus on enhancing networks architecture or increasing the number of images. Notwithstanding, one crucial aspect has not been given strong consideration, the reliability of the annotations.

In this paper, an emotion-in-context dataset reannotation was performed. The participation of at least 15 persons daily, for 20 days, was requested to annotate a subset of the EMOTIC database. An attentiveness promotion (AP) strategy was presented to encourage vigilance of annotators. Likewise, a reliability enhancement (RE) based on the *Z*-Score was applied to keep the most representative annotations per image. These efforts would help diminish imbalances that can be detrimental in further emotion recognition tasks, and increase the reliability of annotations. The new annotation strategy may also reduce the emotion projection effect, which is likely to occur in labeling tasks.

As a result, it was possible to observe that, by decreasing the number of classes and increasing the number of annotators, the interrater accordance rose considerably. Most of the images went from *Fair/Slight*, to *Moderate/Almost Perfect* agreement. Moreover, the number of labeled emotions by image was reduced to four. This is important as it prevents labelings to fall into opposite categories. Finally, qualitatively, it was noted how the annotation went from a multi-labeling with possibly confusing and non-representative categories, to a set of classes highly related to the contents of the images. These results aim at providing trustworthy data for more reliable emotion recognition in context.

An important point to consider is the gender difference in the participants involved in the annotation process. Several studies report that certain factors among labelers, such as sex^29,30^, age^31,32^ or race^33,34^, can lead to a bias in the resulting annotations. In the present work we had a majority of female voters. Consequently, in the near future we aim to compare the current results with those derived from a male majority, as well as balancing the number of voters by gender.

For future research directions, applying the relabeling process to the rest of the EMOTIC database is a hard but worthwhile task. This will lead to the development of a CNN architecture for recognizing emotions in context more robustly. Such emotion classification could lead to useful applications for humans, such as security^35^, education, human-robot interaction, psychological health assessment^36^, affective anomalies discovery and extreme behaviors prevention^37^. Furthermore, it is of our interest to test our newly annotated dataset on state-of-the-art emotion recognition models^14,23,24^.

## Data Availability

The new labelings described in this paper, along with the associated graphs with statistical results, can be accessed through the repositories figshare^27^, Harvard Dataverse^28^ and GitHub (https://github.com/cmtzmiwa/On-Reliability-of-Annotations-in-Contextual-Emotion-Imagery). The subset of images used in this work belongs to EMOTIC. This database is authored by Kosti *et al*.^14^ and is available upon request at https://github.com/rkosti/emotic#access-to-emotic.

## Code availability

The MATLAB 2022 (MathWorks, Inc., Natick, MA, USA) codes developed for getting the results presented in this paper are available at figshare^27^, Harvard Dataverse^28^ and GitHub (https://github.com/cmtzmiwa/On-Reliability-of-Annotations-in-Contextual-Emotion-Imagery).
